# APA GUIDELINES FOR THE EVALUATION OF DEMENTIA: OVERVIEW AND KEY UPDATES

**DOI:** 10.1093/geroni/igac059.792

**Published:** 2022-12-20

**Authors:** Benjamin Mast, Shellie-Anne Levy

**Affiliations:** University of Louisville, Louisville, Kentucky, United States; University of Florida, Gainesville, Florida, United States

## Abstract

This presentation will provide a historical background and a broad overview of the APA Guidelines for the Evaluation of Dementia and Age-Related Cognitive Change. This will begin with an overview of general and procedural guidelines pertaining to dementia assessment and will be followed by an in-depth discussion of key updates in the 2021 revision. The newly revised guidelines recognize racial and cultural disparities in dementia outcomes, and emphasize the importance of multicultural competence in all aspects of dementia assessment. The revised guidelines also integrate person-centered principles into the assessment process and place greater emphasis on understanding the person living with dementia. New guidelines were included to address (1) the assessment of behavioral and psychological changes in the context of dementia, and (2) the importance of assessing the health and well-being of family caregivers.

